# TMAO regulates renal tubular epithelial cell senescence through circVmn2r1

**DOI:** 10.1080/0886022X.2026.2740246

**Published:** 2026-11-09

**Authors:** Lu Zeng, Limin Wei, Shanshan Liang, Lei Chen, Hongli Jiang, Fanfan Gao

**Affiliations:** a Department of Critical Care Nephrology and Blood Purification, The First Affiliated Hospital of Xi’an Jiaotong University, Xi’an, Shaanxi, China; b Department of Aerospace Hygiene, Faculty of Aerospace Medicine, Air Force Medical University, Xi’an, Shaanxi, China; c Department of blood transfusion, the First Affiliated Hospital of Xi’an Jiaotong University, Xi’an, Shaanxi, China; d Department of Obstetrics and Gynecology, the First Affiliated Hospital of Xi’an Jiaotong University, Xi’an, Shaanxi, China

**Keywords:** TMAO, kidney, aging, senescence, circRNA

## Abstract

The gut microbiota-derived metabolite trimethylamine N-oxide (TMAO) is an established risk factor for cardiovascular and renal diseases, yet its role in driving kidney aging remains poorly defined. Circular RNAs (circRNAs) have emerged as critical regulators of cellular senescence, but their integration with systemic metabolic cues in aging processes is largely unknown. Here, we identify a novel signaling axis through which TMAO accelerates renal senescence by dysregulating a specific circRNA pathway. We demonstrate that TMAO treatment induces a senescent phenotype in renal tubular epithelial cells and promotes kidney aging in mice, as evidenced by increased senescence-associated β-galactosidase activity and elevated expression of p53, p21, and p16. Mechanistically, TMAO significantly upregulates the expression of circVmn2r1, a circRNA we previously identified as a key promoter of kidney aging. This upregulation triggers the activation of the the nucleotide-binding domain, leucine-rich repeat-containing family, pyrin domain-containing 3 (NLRP3) inflammasome, leading to increased caspase-1 cleavage and elevated levels of the pro-inflammatory cytokines pro-IL-1β and pro-IL-18. Crucially, genetic knockdown of circVmn2r1 *in vitro* and *in vivo* significantly attenuates TMAO-induced NLRP3 upregulation, inflammasome activation, and cellular senescence. Our findings reveal that TMAO functions as an upstream metabolic trigger that promotes renal aging *via* the circVmn2r1/NLRP3 inflammasome axis. This study elucidates a direct link between a diet-derived metabolite and a specific circRNA-mediated pro-aging pathway, identifying circVmn2r1 as a potential therapeutic target for mitigating metabolic stress-induced kidney aging.

## Graphical Abstract

**Figure:**
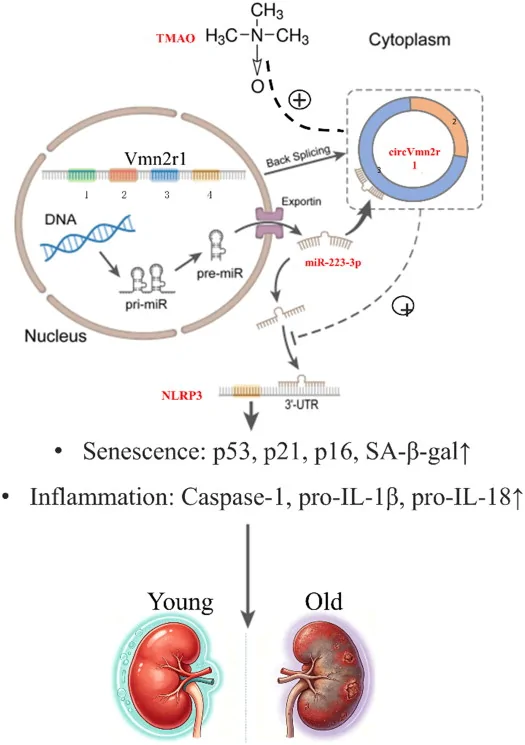

## Introduction

Kidney aging is a critical biological process that not only accelerates the progression of acute and chronic kidney diseases but also contributes to systemic pathologies in distant organs [1–4]. Consequently, elucidating the molecular drivers of renal aging is paramount for developing strategies to promote healthy aging and mitigate age-related renal decline. Recent studies have identified ACSS2-mediated histone crotonylation as a driver of renal fibrosis *via* IL-1β, and FFAR4 signaling as a protective axis against podocyte injury and tubular senescence [5–7]. Among the various regulatory molecules, circular RNAs (circRNAs)—stable, covalently closed non-coding RNA transcripts—have emerged as vital players in aging and age-related diseases [8–10]. However, their specific biological functions and mechanistic underpinnings in kidney aging remain largely unexplored. Uncovering these roles could illuminate novel diagnostic and therapeutic avenues.

Our previous research identified circVmn2r1 as a key circRNA implicated in kidney aging [11]. RNA sequencing revealed it to be one of the most abundant circRNAs in mouse kidney, with expression significantly elevated in 24-month-old mice compared to 3-month-old counterparts. Functional studies demonstrated that overexpression of circVmn2r1 accelerated kidney aging *in vivo* in senescence-accelerated mouse prone 8 (SAMP8) models. At the cellular level, gain- and loss-of-function experiments in mouse renal tubular epithelial (TCMK-1) cells established that circVmn2r1 inhibits proliferation and promotes senescence. Mechanistically, we delineated a competing endogenous RNA (ceRNA) network wherein circVmn2r1 functions as a molecular sponge for miR-223-3p, thereby relieving the microRNA’s repression of its target, the nucleotide-binding domain, leucine-rich repeat-containing family, pyrin domain-containing 3 (NLRP3)—a key inflammasome component itself capable of driving senescence in TCMK-1 cells. These findings positioned the circVmn2r1/miR-223-3p/NLRP3 axis as a significant promoter of kidney aging [8,9,12]. Despite this progress, a crucial upstream question persists: what physiological or pathological trigger initiates the dysregulation of this pro-aging circuit? In this context, trimethylamine N-oxide (TMAO), a gut microbiota-derived metabolite, has garnered substantial attention. Elevated circulating TMAO is a recognized risk factor for cardiovascular and renal diseases and is associated with inflammation and oxidative stress—hallmarks of aging [13,14]. Healthy individuals typically have plasma TMAO <10 µM, while chronic kidney disease (CKD) patients exhibit levels ranging from 20 to 200 µM or even higher [15]. Intriguingly, TMAO has been shown to activate the NLRP3 inflammasome in various cell types [16,17]. This connection raises a compelling hypothesis: could TMAO serve as an endogenous metabolic trigger that promotes renal aging by activating the previously identified circVmn2r1/NLRP3 pathway? The potential link between this diet-derived metabolite and a specific circRNA-regulated aging mechanism remains entirely uninvestigated.

Therefore, building upon our prior discovery of the circVmn2r1-driven aging pathway, this study aims to investigate TMAO as an upstream regulator. We hypothesize that TMAO promotes renal cellular and tissue senescence by upregulating circVmn2r1, which in turn activates the NLRP3 inflammasome pathway. To test this, we will [1]: confirm the senescent effects of TMAO on renal tubular cells and in mouse kidney [2]; examine whether TMAO upregulates circVmn2r1 expression both *in vitro* and *in vivo* [3]; verify the associated activation of the NLRP3 inflammasome and inflammatory cascade; and [4] definitively establish the functional necessity of circVmn2r1 in mediating TMAO’s effects through rescue experiments. This work seeks to bridge a critical knowledge gap by identifying a diet-microbiome-metabolite axis that accelerates kidney aging *via* a defined circRNA mechanism, offering potential novel targets for intervention.

## Materials and methods

### Preparation of trimethylamine N-oxide (TMAO)

Trimethylamine N-oxide dihydrate (TMAO, Sigma-Aldrich, Cat 317594, purity ≥95%) was purchased from Sigma-Aldrich (St. Louis, MO, USA). TMAO dihydrate stock solution (5 M in water) was filter-sterilized using a 0.22 μm syringe filter, aliquoted, and stored at −20 °C. Working solutions for cell culture or drinking water were freshly prepared from a freshly thawed aliquot each time. Drinking bottles were made of amber glass to protect from light.

### Cell culture and treatment

Mouse renal tubular epithelial cells (TCMK-1) (ATCC, USA) were cultured in Dulbecco’s Modified Eagle Medium supplemented with 10% (v/v) fetal bovine serum and 1% penicillin-streptomycin (Gibco) at 37 °C in a humidified 5% CO_2_ incubator. Cells were seeded in appropriate culture plates and allowed to adhere overnight. For TMAO treatment, cells were incubated with fresh medium containing specified 100 mM concentrations of TMAO for designated time periods (24, 48, 72 h). Control groups received an equivalent volume of PBS vehicle.

### Animal experiments

All animal procedures were approved by the Institutional Animal Care and Use Committee (IACUC) of Xi’an Jiaotong University (Approval No. 2018-G-164) and conducted in accordance with the National Institutes of Health Guide for the Care and Use of Laboratory Animals. Eight-week-old male C57BL/6J mice were purchased from Xi’an Jiaotong University and housed under standard conditions (12 h light/dark cycle, 22 ± 2 °C, 50 ± 10% humidity) with free access to food and water. After one week of acclimatization, mice were randomly assigned to experimental groups (*n* = 6 per group). For TMAO treatment, TMAO was given in drinking water at a concentration of 1.5% for 16 weeks and renewed every other day [18]. Mice were deeply anesthetized by intraperitoneal injection of chloral hydrate (300–400 mg/kg body weight) and euthanized by cervical dislocation. Death was confirmed by the absence of respiration and heartbeat. Kidneys were rapidly harvested, weighed, and divided for analysis: one portion was fixed in 10% neutral buffered formalin for histology, another was snap-frozen in liquid nitrogen for molecular biology assays, and a third portion was embedded in OCT compound for frozen sectioning.

### Cell viability assay (CCK-8)

Cell viability was assessed using the Cell Counting Kit-8 (CCK-8, Dojindo, Japan) according to the manufacturer’s instructions. Briefly, TCMK-1 cells were seeded in 96-well plates and treated as indicated. At the end of treatment, 10 μL of CCK-8 reagent was added to each well, followed by incubation at 37 °C for 2 h. Absorbance was measured at 450 nm using a microplate reader (BioTek, USA). Cell viability was expressed as a percentage relative to the control group.

### Senescence-Associated β-galactosidase (SA-β-gal) staining

Cellular senescence was detected using the Senescence β-Galactosidase Staining Kit (Cell Signaling Technology, USA) following the manufacturer’s protocol. After treatment, cells (or cryosections of kidney tissue) were fixed, washed, and incubated with the SA-β-gal staining solution overnight at 37 °C in a dry incubator without CO_2_. Senescent cells were identified by the development of blue cytoplasmic staining under a light microscope. The percentage of SA-β-gal-positive cells was quantified from at least 3 random fields per sample.

### Gene silencing with small interfering RNA (siRNA)

To knockdown circVmn2r1 expression, TCMK-1 cells were transfected with specific siRNAs targeting the back-splice junction of circVmn2r1 or a non-targeting control siRNA (si-NC) using Lipofectamine 3000 reagent (Invitrogen, USA) in Opti-MEM medium according to the manufacturer’s protocol. After 6 h, the transfection medium was replaced with complete growth medium. For *in vivo* knockdown, circVmn2r1-specific or control siRNAs (Shanghai Gene Era Bio-Science, Shanghai, PRC) were administered to mice *via* tail vein injection twice weekly for 4 weeks, the whole process were similar with previous published paper [11]. Transfection efficiency was confirmed by qRT-PCR.

### Quantitative Real-Time PCR (qRT-PCR)

Total RNA was extracted from cells or homogenized kidney tissues using TRIzol reagent (Invitrogen, USA). RNA quality and concentration were assessed by spectrophotometry. The A260/280 ratio of sample is within the range of 1.8 to 2.0, which indicates that the extraction quality is qualified. For circVmn2r1 detection, total RNA was first treated with RNase R (Epicentre, USA) to degrade linear RNAs. Complementary DNA (cDNA) was synthesized using the PrimeScript^™^ RT reagent Kit (Takara, Japan). Quantitative PCR was performed using SYBR Green Premix Pro Taq HS (Accurate Biology, China) on Real-Time PCR System (Applied Biosystems, USA). Glyceraldehyde-3-phosphate dehydrogenase (GAPDH) was used as an internal control. Relative gene expression was calculated using the 2^(-ΔΔCt) method. circVmn2r1-1 primer: F: ACCTACCTTTGATTTCTGGCAGGTT, R: TGGTAGCCCAGAGTATGGTTGGG; si-circVmn2r1-1 primer: F: UUGAUUUCUGGCAGGUUUATT, R: UAAACCUGCCAGAAAUCAATT.

### Western blot analysis

Total protein was extracted from cells or kidney tissues using RIPA lysis buffer containing protease and phosphatase inhibitors. Protein concentration was determined with a BCA Protein Assay Kit (Pierce, USA). Equal amounts of protein (30 μg) were separated by SDS-PAGE and transferred onto PVDF membranes (Millipore, USA). Membranes were blocked with 5% nonfat milk and incubated overnight at 4 °C with primary antibodies against: p53 (ab179477, Abcam, 1:1000), p21 (ab109199, Abcam, 1:1000), p16 (ab108349, Abcam, 1:1000), NLRP3 (15101S, CST, 1:1000), caspase-1 (2225S, CST, 1:1000), pro-IL-1β (16806-1-AP, Proteintech, 1:1000), pro-IL-18 (57058, CST, 1:1000), GAPDH (10494-1-AP, Proteintech, 1:5000). After incubation with appropriate HRP-conjugated secondary antibodies, protein bands were visualized using an enhanced chemiluminescence (ECL) substrate (Millipore, USA) and imaged with a chemiluminescence imaging system (Tanon, China). Band intensity was quantified using ImageJ software (NIH, USA).

### Immunofluorescence staining

Cells grown on coverslips or frozen kidney sections were fixed with 4% paraformaldehyde, permeabilized with 0.1% Triton X-100, and blocked with 5% bovine serum albumin (BSA). Samples were incubated overnight at 4 °C with primary antibody against NLRP3 (1:200, Cell Signaling Technology), followed by incubation with a fluorophore-conjugated secondary antibody (Alexa Fluor^®^ 488, Invitrogen) for 1 h at room temperature in the dark. Nuclei were counterstained with DAPI (4′,6-diamidino-2-phenylindole). Images were captured using a confocal laser scanning microscope (Leica, Germany).

### Statistical analysis

All experiments were performed with at least three independent biological replicates and three technical replicates for each group. All data are presented as mean ± standard deviation (SD) from at least three independent experiments. Statistical analysis was performed using GraphPad Prism software (version 9.0). Differences between two groups were analyzed using an unpaired two-tailed Student’s t-test. Comparisons among multiple groups were analyzed by one-way analysis of variance (ANOVA) followed by Tukey’s *post hoc* test. A p-value of less than 0.05 was considered statistically significant. * *p* < 0.05, ** *p* < 0.01, *** *p* < 0.001.

## Results

### Effect of TMAO on TCMK-1 Cell viability

The impact of TMAO on the viability of TCMK-1 cells was assessed using the Cell Counting Kit-8 (CCK-8) assay. Cells were treated with varying concentrations of TMAO for different durations. Cell viability progressively decreased with increasing TMAO concentrations at a fixed treatment time. Conversely, prolonging the treatment duration at a constant TMAO concentration also led to a gradual reduction in proliferative capacity (Figure 1A).

**Figure 1.**
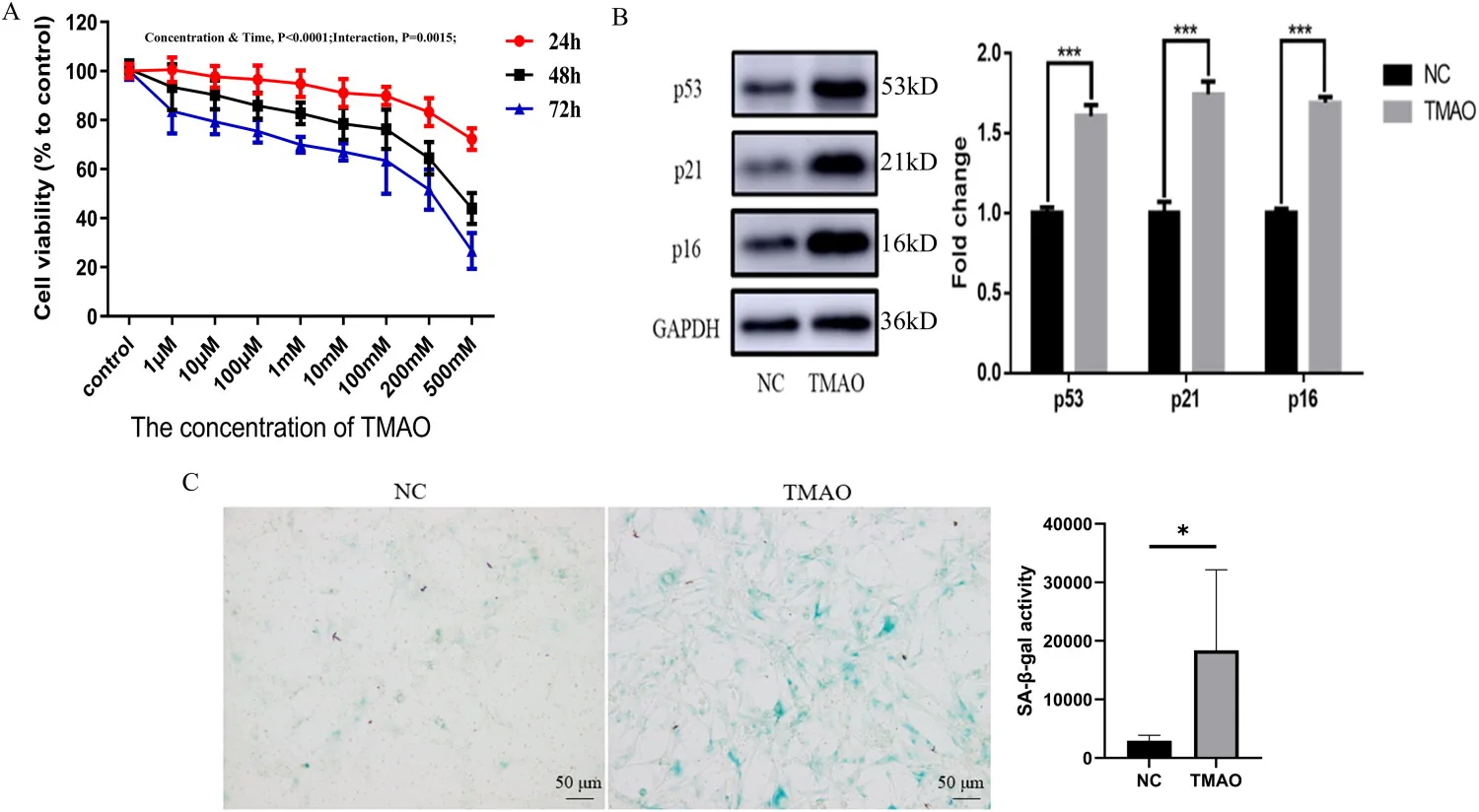
TMAO inhibits TCMK-1 cell viability in a concentration- and time-dependent manner and promotes cellular senescence. (A) TCMK-1 cells were treated with increasing concentrations of TMAO (1, 10, 200, 500 mM) for 24, 48, or 72 h. Cell viability was assessed by CCK-8 assay. Data are presented as mean ± SD. Data analyzed by two-way ANOVA. Row factor (TMAO concentration) accounted for 60.77% of total variation (*P* < 0.0001, ****), column factor (treatment time) accounted for 25.35% variation (*P* < 0.0001, ****), and row–column interaction accounted for 6.49% variation (*P* = 0.0015, **). (B) TCMK-1 cells were treated with 100 mM TMAO for 72 h. Representative Western blot images show protein levels of senescence-associated markers p53, p21, and p16; (C) Representative SA-β-gal images of TCMK-1 cells after treatment with 100 mM TMAO for 72 h. All data are representative of three independent experiments.

### TMAO induces cellular senescence in TCMK-1 cells

To investigate whether TMAO promotes cellular senescence, TCMK-1 cells were stimulated with 100 mM TMAO for 72h. Western blot analysis revealed a significant upregulation of key senescence-associated markers, including the tumor suppressor p53 and its downstream effector p21, as well as the cyclin-dependent kinase inhibitor p16 (Figure 1B). Consistent with this molecular profile, senescence-associated β-galactosidase (SA-β-gal) activity was markedly enhanced upon TMAO treatment, as visualized by increased staining (Figure 1C). These findings collectively indicate that TMAO induces a senescent phenotype in TCMK-1 cells.

### TMAO upregulates circVmn2r1, NLRP3, and pro-inflammatory mediators in TCMK-1 cells

We next examined the effect of TMAO on specific molecular pathways. Quantitative real-time PCR (qRT-PCR) analysis showed that TMAO stimulation significantly elevated the expression level of the circular RNA circVmn2r1 in TCMK-1 cells (Figure 2A). Immunofluorescence staining demonstrated a substantial increase in the protein expression of NLRP3, a core component of the inflammasome, following TMAO treatment (Figure 2B). Furthermore, western blotting results indicated that TMAO triggered the activation of the NLRP3 inflammasome pathway, evidenced by a significant increase in the cleaved, active form of caspase-1 and the precursor forms of the inflammatory cytokines IL-1β (pro-IL-1β) and IL-18 (pro-IL-18) (Figure 2C). These results suggest that TMAO promotes inflammatory responses in TCMK-1 cells, potentially linked to circVmn2r1 upregulation and NLRP3 inflammasome activation.

**Figure 2.**
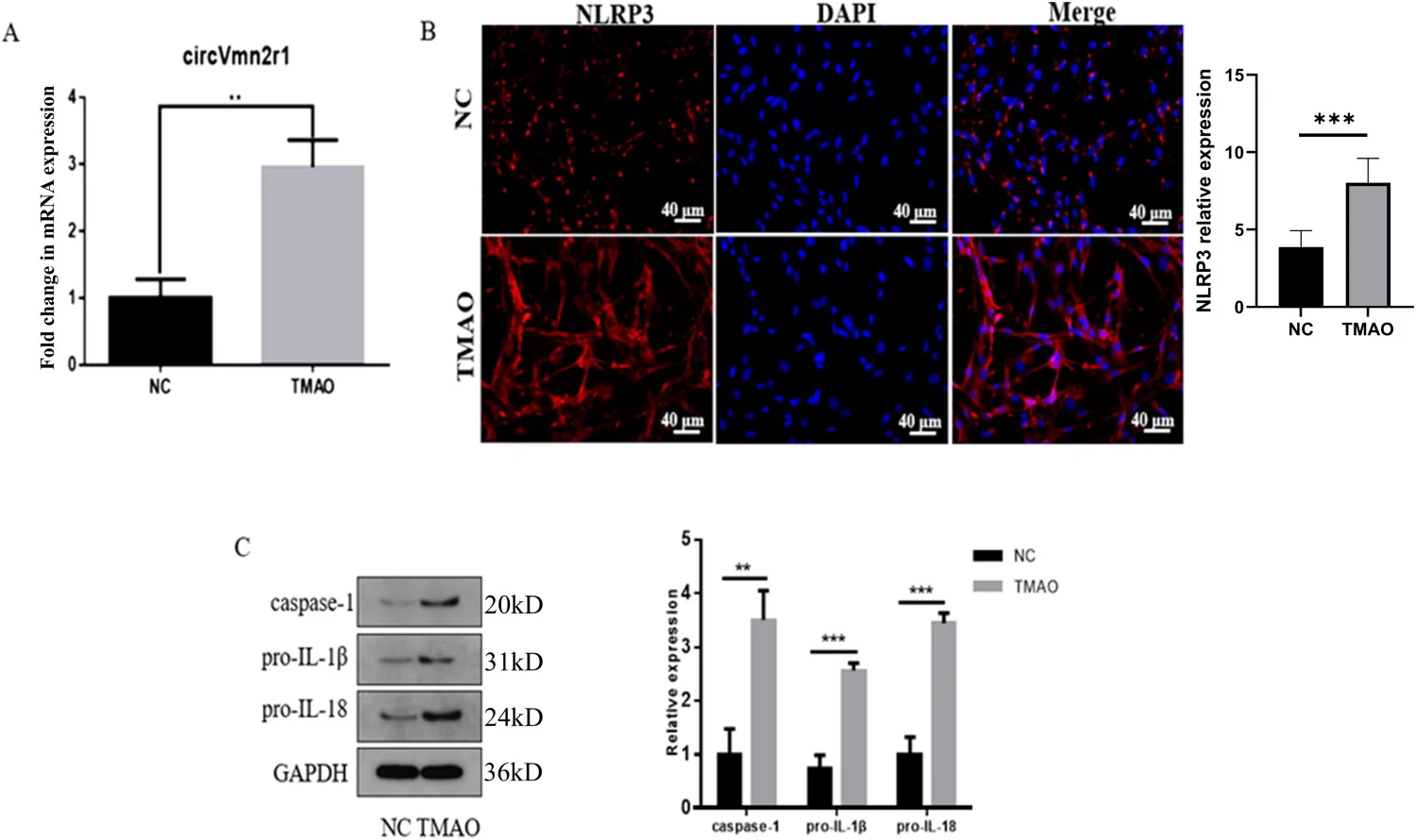
TMAO increases the expression of circVmn2r1, NLRP3, and inflammasome-related factors in TCMK-1 cells. (A) TCMK-1 cells were treated with 100 mM TMAO for 72 h. The expression level of circVmn2r1 was measured by qRT-PCR. (B) Immunofluorescence staining of NLRP3 (red) and DAPI (blue) in TCMK-1 cells after treatment with 100 mM TMAO for 72 h. Scale bar = 40 μm. (C) Western blot analysis of inflammasome-related proteins (caspase-1, pro-IL-1β, pro-IL-18) in TCMK-1 cells treated with 100 mM TMAO for 72 h. All data are representative of three independent experiments.

### Knockdown of circVmn2r1 attenuates TMAO-induced senescence and NLRP3 upregulation

To determine the functional role of circVmn2r1 in mediating TMAO’s effects, we performed loss-of-function experiments. TCMK-1 cells were transfected with either a non-targeting control siRNA (si-NC) or a circVmn2r1-specific siRNA (si-circVmn2r1), followed by treatment with or without TMAO. Western blotting analysis of key proteins showed that TMAO stimulation significantly increased the expression of p53, p21, and NLRP3 in the si-NC group. In contrast, knockdown of circVmn2r1 alone reduced their basal expression. Importantly, in cells with circVmn2r1 knockdown, the pro-senescent and pro-inflammatory effects of TMAO were markedly attenuated, as the upregulation of p53, p21, and NLRP3 was significantly blunted (Figure 3). These results indicate that circVmn2r1 knockdown can partially attenuate the TMAO-induced upregulation of NLRP3 and cellular senescence, suggesting that TMAO promotes senescence in TCMK-1 cells primarily by upregulating circVmn2r1.

**Figure 3.**
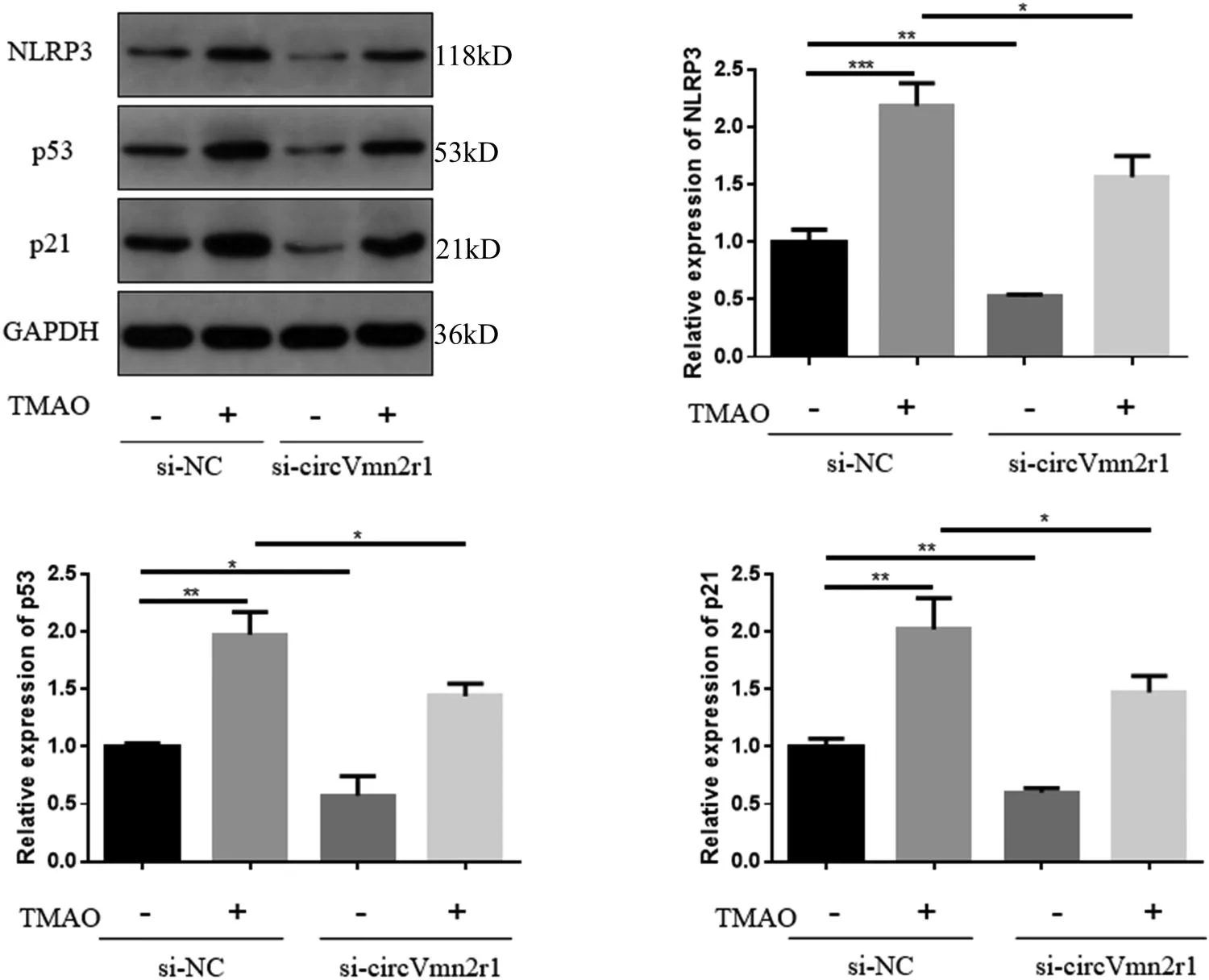
circVmn2r1 mediates the pro-senescent effects of TMAO on TCMK-1 cells. Representative Western blot images showing protein levels of NLRP3, p53, and p21. All data are representative of three independent experiments.

### TMAO promotes renal senescence in mice

To validate our *in vitro* findings *in vivo*, C57BL/6J mice were administered TMAO dissoving in drinking water at a concentration of 1.5% for 16 weeks. Western blotting analysis of kidney tissue lysates revealed a significant increase in the protein levels of senescence markers p53, p21, and p16 in the TMAO-treated group compared to the control group (Figure 4A). This was corroborated by enhanced SA-β-gal staining activity in the kidney sections from TMAO-treated mice (Figure 4B), confirming that TMAO induces renal senescence *in vivo*.

**Figure 4.**
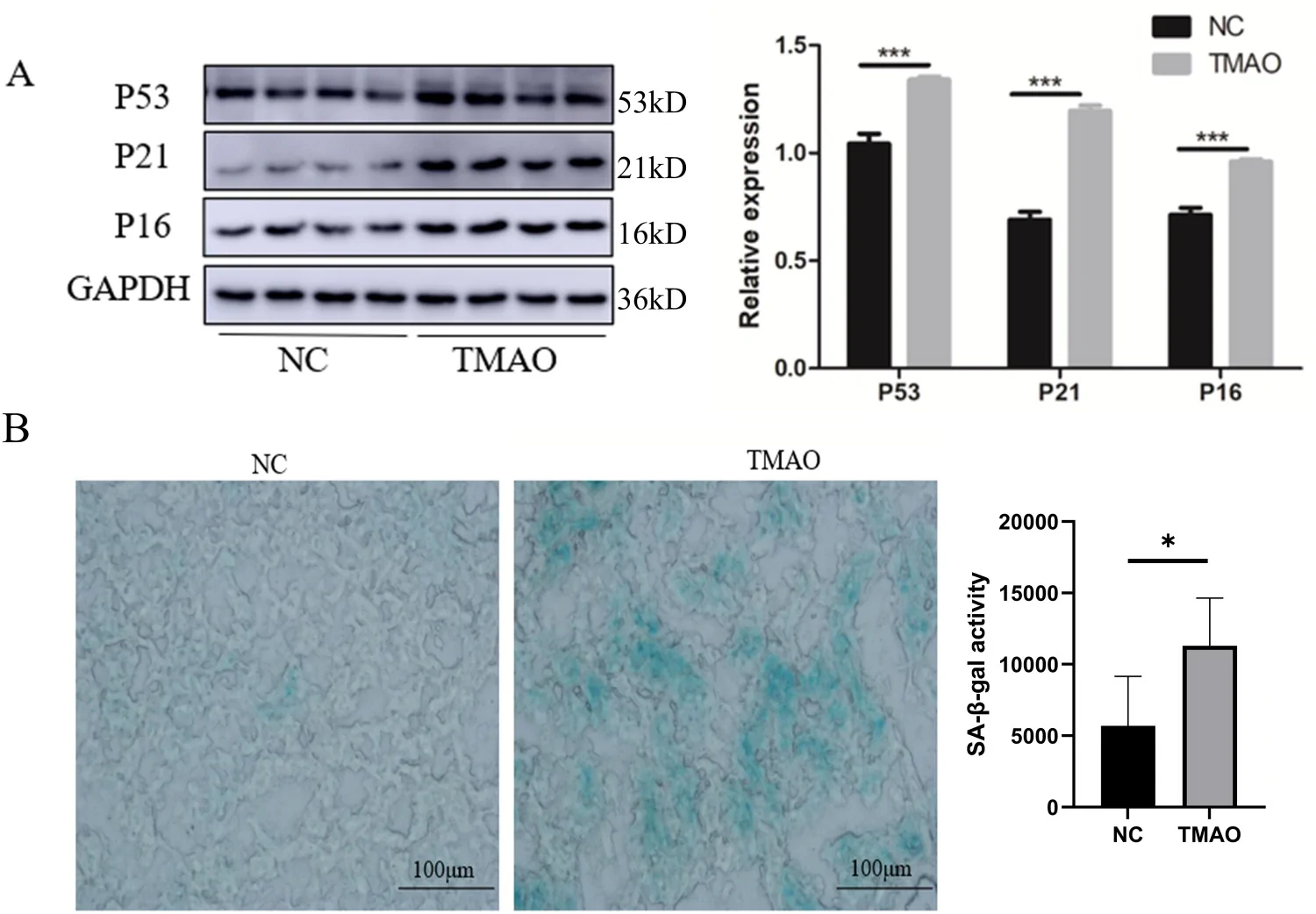
TMAO promotes kidney senescence in mice. (A) Representative Western blot images showing protein levels of senescence markers p53, p21, and p16 in mouse kidney. (B) Representative SA-β-gal images of mouse kidney sections in different group (scale bar = 100 μm). Representative blots/images are shown; quantitative data are derived from *n* = 4 mice per group.

### TMAO elevates circVmn2r1, NLRP3, and inflammatory factors in mouse kidney

Consistent with the cellular data, qRT-PCR analysis showed a significant upregulation of circVmn2r1 expression in the kidneys of TMAO-treated mice (Figure 5A). Immunofluorescence analysis indicated a marked increase in NLRP3 protein expression within the kidney tissue (Figure 5B). Moreover, western blotting demonstrated elevated levels of active caspase-1, pro-IL-1β, and pro-IL-18 in the TMAO group (Figure 5C). These results confirm that TMAO similarly activates circVmn2r1 expression, the NLRP3 inflammasome, and associated inflammatory pathways in the mouse kidney.

**Figure 5.**
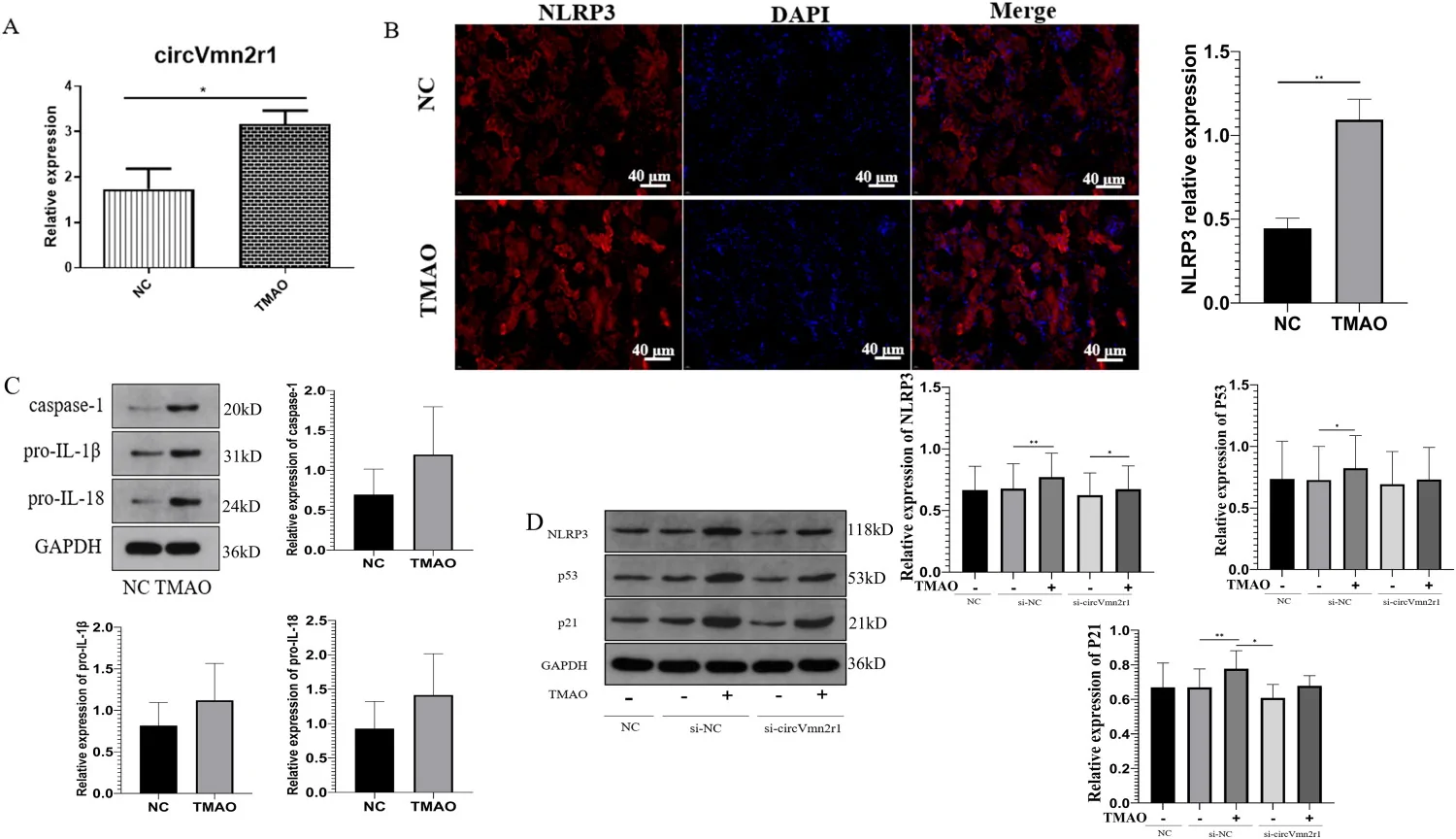
TMAO upregulates circVmn2r1, NLRP3, and inflammatory factors in mouse kidney; knockdown of circVmn2r1 attenuates TMAO-induced renal senescence. (A) Relative expression of circVmn2r1 in mouse kidney measured by qRT-PCR. (B) Immunofluorescence staining of NLRP3 (red) and DAPI (blue) in mouse kidney sections. Scale bar = 40 μm. (C) Western blot analyses of kidney inflammasome-related proteins (NLRP3, caspase-1, pro-IL-1β, pro-IL-18) and senescence markers (p53, p21) in control and TMAO treatment mice group. (D) Western blot analyses of NLRP3, p53, and p21 of mice kidney after treatment with TMAO together with si-NC or si-circVmn2r1. Representative blots/images are shown; quantitative data are derived from *n* = 3 mice per group.

### Inhibition of circVmn2r1 suppress TMAO-induced renal senescence *In vivo*

To investigate the *in vivo* role of circVmn2r1, mice were subjected to different treatments involving TMAO administration and *in vivo* delivery of siRNAs targeting circVmn2r1. Western blotting analysis of kidney tissues showed that TMAO treatment significantly increased the expression of p53, p21, and NLRP3. Conversely, knockdown of circVmn2r1 alone reduced their expression. Most notably, in mice receiving both circVmn2r1 siRNA and TMAO, the upregulating effects of TMAO on these senescence and inflammation markers were significantly mitigated (Figure 5D). This demonstrates that circVmn2r1 is a critical mediator *in vivo*, and its inhibition can suppress the pro-senescent and pro-inflammatory effects of TMAO in the kidney.

## Discussion

Uremic toxins originate from intestinal flora are harmful for human health [19]. When dysbiosis of gut microbial homeostasis appears, pathogenic uremic toxins may increase and one of them is TMAO [20]. TMAO has been proved to be associated with impaired renal function [21], the concentration of TMAO is increased in hemodialysis (HD) patients, compared to healthy subjects [22]. TMAO can induce brain aging and age-related cognitive dysfunction [18], also it accelerates endothelial cell senescence and vascular aging [23]. Kidney senescence belongs to the pre-stage of acute and chronic kidney diseases [24,25]. It has been shown that the gut microbiota dysbiosis was related to kidney senescence [26,27], but the direct evidences about TMAO promoting renal senescence are still rare.

Our research observed both in renal tubular epithelial cells *in vitro* and in the kidney tissue of mice exposed to chronic TMAO administration *in vivo*, that TMAO administration successfully recapitulated the hallmarks of renal aging—increased SA-β-gal activity and elevated p53/p21/p16 expression. This suggests that elevated TMAO levels, as seen in certain dietary patterns or dysbiosis, could accelerate renal aging. It is consistent with previous research results [28], potentially explaining part of the link between diet, the gut microbiome, and the progression of age-related kidney dysfunction.

Systemic metabolic signals may promote disease progression through interacting with intracellular ceRNA networks [29,30]. Our study demonstrates that TMAO, functions as an upstream physiological trigger that promotes cellular and tissue aging by upregulating circVmn2r1, thereby activating the NLRP3 inflammasome cascade. Most importantly, *in vivo* inhibition of circVmn2r1 mitigated TMAO-induced renal damage, highlighting the translational potential of targeting this node. This finding is significant because it links a well-established circulating risk factor for cardiorenal disease [22,31] to the dysregulation of a specific non-coding RNA with proven pro-aging functions. Our findings must be interpreted in the context of our prior research, which established the foundational role of the circVmn2r1/miR-223-3p/NLRP3 axis in kidney aging. We previously identified circVmn2r1 as an age-upregulated circRNA in mouse kidney, demonstrated its functional capacity to accelerate renal aging *in vivo*, and delineated its mechanistic action as a molecular sponge for miR-223-3p, leading to the derepression and upregulation of its target, NLRP3 [11]. The current study builds directly upon this framework by addressing a pivotal upstream question: what factors initiate the dysregulation of this pro-aging pathway? We identified TMAO as a key endogenous metabolic signal capable of inducing this specific cascade. This will advance our understanding of kidney aging by identifying a novel diet-microbiome-metabolite axis that activates a specific circRNA-regulated pathway to drive renal senescence.

Several limitations should be acknowledged. First, while we establish TMAO as an inducer of circVmn2r1, the precise transcriptional or post-transcriptional mechanism by which it upregulates this circRNA requires further investigation. Second, the *in vivo* siRNA-mediated knockdown, while effective, is transient; future studies using genetic models with conditional circVmn2r1 deletion would provide more definitive proof. Third, our study does not provide data on whether TMAO-induced tubular senescence translates into measurable kidney functional decline, future studies should include functional endpoints. Fourth, our study focuses on a single circRNA-metabolite interaction; the broader landscape of TMAO-regulated non-coding RNAs in the kidney remains an open and important field for exploration.

In conclusion, this study elucidates a novel pathological circuit wherein the gut metabolite TMAO accelerates kidney aging by activating the circVmn2r1/miR-223-3p/NLRP3 axis. These findings position circVmn2r1 not only as a mediator of aging but also as a sensor of metabolic derangement, linking systemic physiology to cellular aging pathways. Intervening in the TMAO-circVmn2r1-NLRP3 axis may therefore represent a promising dual-purpose strategy to slow renal aging and mitigate the progression of age-related kidney diseases associated with elevated TMAO.

## Data Availability

The data that support the findings of this study are available from the corresponding author, upon reasonable request.
